# Supporting Population Mental Health in the Wake of Mass Tragedies

**DOI:** 10.5694/mja2.70160

**Published:** 2026-03-08

**Authors:** Susan J. Rees, Derrick M. Silove

**Affiliations:** ^1^ UNSW Sydney Sydney New South Wales Australia

**Keywords:** community psychiatry, post‐traumatic stress disorder, trauma and stressor related disorders

## Abstract

The Bondi Beach terrorist attack has caused widespread community distress resulting in complex psychosocial challenges that require urgent attention to protect population mental health. Acute reactions including shock, fear and anger are normative, while the psychological responses unfolding over time may vary from adaptive to dysfunctional. A range of mental health and psychosocial services have been mobilised to address acute needs; however, to ensure a sustained and coherent framework of care, interventions should be guided by a set of principles that accurately respond to the unique nature of the event. We provide an overview of the Adaptation and Development After Persecution and Trauma (ADAPT) model as an overarching framework that can guide psychosocial interventions and promote adaptive coping, recovery and resilience.

## 1 Introduction

The December 2025 Bondi Beach terrorist attack has left an aftermath of collective distress and created psychosocial challenges that demand attention to safeguard long‐term mental health [1, 2, 3]. Intentional human acts of mass violence, in this instance reportedly motivated by extremist religious and racist ideologies [4], are particularly injurious to the psychological well‐being of the target Jewish population in toto, family members and friends of the deceased, those injured and who witnessed injury and death, first responders and others who provided assistance. The impact of the Bondi attack is heightened by the timing and location, being perpetrated against a gathering of people celebrating Chanukah, a festival memorialising the liberation of the Jewish people from persecution. The location of the attack was particularly poignant, occurring at Bondi Beach, renowned nationally and internationally as a symbol of Australia's traditionally peaceable and harmonious way of life.

## 2 The Adaptation and Development After Persecution and Trauma (ADAPT) Model

In the acute phase following a mass trauma event motivated by intentional human violence, it is normative for impacted populations to experience a gamut of emotional reactions, including shock, confusion, fear and anger [5, 6, 7]. As time passes, psychological responses will persist or emerge, ranging from adaptive to dysfunctional. Following the Bondi attack, various front‐line psychosocial and specialist services have been mobilised to address acute mental health needs. It is, however, essential that all contributors work together, and with an appropriate and theoretically selected set of psychosocial principles to guide and deliver programmes that will support sustained recovery across individual and community levels of the society [8, 9]. The ADAPT model identifies five interrelated psychosocial pillars on which stable societies are grounded, and which are disrupted by intentional acts of mass violence such as that which occurred at Bondi: (i) safety and security, (ii) attachment and bonds, (iii) justice, (iv) identities and roles, and (v) existential meaning [9, 10, 11].

The model assumes that mass violence evokes universal psychobiological defensive responses although the form, severity and course of these reactions are shaped by the historical, social, cultural and political context in which the trauma occurs. Trauma responses appear along a continuum from normative to severe, are heterogeneous in their expression, and dependent on individual differences in vulnerability and resilience. Coordinated care, involving a range of psychosocial and specialist mental health services, is therefore required to meet the diversity of needs. Recovery should aim not only to restore well‐being but also to identify and strengthen individual and community resilience to respond to future trauma [8, 9, 10, 12]. In this context, Jewish people in the diaspora possess many strengths, including strong social networks, established religious institutions, shared spiritual and cultural systems of meaning, high levels of education, and a history of adaptive coping even in the face of extreme past persecution, most notably the Holocaust [13].

## 3 Pillar 1: Safety and Security

Pillar 1 of the ADAPT model identifies the domain of safety and security [9, 10]. Humans are biologically equipped by evolution to respond to mortal threats (to themselves, their families and their communities) by instantaneously mobilising the fight, flight and/or freeze response. Following mass trauma, it is normal for survivors to feel disorientated, confused and numb, and/or to feel over‐aroused and hypervigilant to further attack, with associated physical symptoms (shakiness, weakness, rapid heart rate, difficulty breathing and abdominal upsets) [6, 14]. Interventions should include practical assistance and counselling focused on immediate needs and concerns, consistent with the principles of the widely applied psychological first aid model [15]. At the same time, there is a consensus that implementing systematic trauma debriefing in which counsellors encourage repeated rehearsal of trauma memories is not recommended in the acute phase [16].

Although post‐traumatic stress disorder (PTSD) is the specific mental health response associated with mass trauma, survivors can manifest a wide range of disorders, including depression, severe forms of anxiety and, in some individuals, drug or alcohol misuse. Moreover, persons with pre‐existing mental health disorders can experience a relapse or worsening of symptoms. The typical responses of the more severe forms of PTSD include intrusive memories in the form of nightmares and flashbacks, avoidance of trauma reminders, marked social withdrawal and extreme hyperarousal, including exaggerated startle responses, fearfulness, hypervigilance and insomnia [6, 14]. Those at highest risk of PTSD include people directly exposed to the violence and their families, women and children and, at a population level, the substantial number of individuals with past histories of trauma or pre‐existing mental illness [17]. Moreover, deliberate human‐engendered violence [7] of the type enacted at Bondi is more likely to result in the complex form of PTSD, especially in those with a prior history of interpersonal trauma [18]. In these persons, difficulty regulating emotions, interpersonal problems, and a disturbed sense of self in the form of guilt and shame are prominent features [18]. Where PTSD symptoms are severe or complex, referral for a comprehensive assessment via primary care to specialist mental health services is indicated [6, 18].

More generally, it is expected that the targeted Jewish community will experience difficulties restoring a sense of communal safety, especially given their long history of persecution (noting that tragically, one of those killed at Bondi was a survivor of the Holocaust) [19, 20]. In addition, the notable upsurge in antisemitism involving physical and property‐targeted attacks, including against synagogues, graffiti, harassment and hate speech, only serves to sensitise the community to fear of further attack [21, 22].

There is also a heightened sense of insecurity experienced by other minority groups, particularly Muslim and Arab Australians. These groups have experienced substantial mental distress since the recent Middle East conflict [23], and an increase in ethnic and religiously motivated attacks and threats to people and property, including to mosques, following the Bondi terror incident [23, 24, 25]. It is therefore pertinent to recognise that there is a more generalised heightening of fear and risk of inter‐communal tensions in Australia's multicultural society.

Restoring a durable sense of safety is a complex, multilevel task requiring not only tangible and cognitive reassurance but also attention to deep emotional, symbolic and psychophysiological responses to perceived threat [26]. For the Jewish population, protection of community facilities (places of worship, schools and places of gathering) is essential to confer feelings of safety [9, 26]. In times of crisis such as this, it is vital therefore that political leaders of all persuasions speak out in unison to reassure the nation, defend intercultural tolerance and uphold our shared values of peace, respect and diversity [23, 24, 27, 28].

## 4 Pillar 2: Attachments and Bonds

Pillar 2 identifies the domain of attachments and bonds as a necessary foundation of a society recovering after mass trauma. The violent loss of family, friends and fellow Australians, as occurred at Bondi Beach, inevitably generates widespread feelings of grief, an expected, normative response both at an individual and collective level, but one that is intensified by the historical and contemporary context given the repeated loss by mass violence and persecution experienced by Jewish people across the generations [19, 20, 29].

Communal activities that support stabilisation of grief reactions include memorial services and vigils, where religious, community and political leaders help the population draw on their faith, sense of common history and traditions to provide solace. Support from family, friends and, where necessary, counsellors working in front‐line social agencies will play a key role in stabilisation for the bereaved, recognising that, for some, grief is a process that may never be fully resolved [6, 29].

A minority of people will develop overwhelming and persisting grief reactions in which symptoms often overlap with those of PTSD, although in these cases the emphasis is on preoccupations with and feelings of yearning for the lost person [14, 17, 29]. This subgroup will require professional help from specialist mental health professionals who are experienced in providing psychotherapy for prolonged and complicated grief reactions and the risk of interrelated depression and anxiety [6, 29].

## 5 Pillar 3: Justice

Pillar 3 identifies the domain of justice, a key issue for mass terrorist attack survivors and their communities. Anger and frustration are common responses to perceived injustice and the sense of being senselessly victimised, a central element of trauma that lies at the core of a terrorist attack [14, 30]. In relation to the Jewish community, these feelings will be intensified by the contemporary upsurge in antisemitism across the Australian society [21, 22, 27]. In that sense, collective anger needs to be recognised as a multidimensional reaction, which is understandable in the historical context of the Jewish community's experience of multigenerational trauma. At the same time, loss of control of anger can be a source of extreme distress for the individual survivor and their family members, resulting in major functioning difficulties and risk of interpersonal conflict [30]. For these persons, professional assistance may be necessary to ensure stabilisation of their severe trauma reactions.

## 6 Pillar 4: Identities and Roles

Pillar 4 refers to the importance of identities and roles, a domain that can be fundamentally undermined by acts of terrorism. Individuals can lose confidence in their feelings of belonging within the wider society, noting that in the instance of the Jewish population after the Bondi terror attack, there are reports of individuals feeling obliged to conceal their identities for fear of antisemitic victimisation [19, 21]. The recent history of conflict and human rights violations in the Middle East, and the political polarisations that have been accentuated nationally and internationally, only add to this sense of vulnerability related to Jewish identity in the diaspora. It is significant that the fragmentation of a sense of identity and the loss of roles that promote mental health are key features of a severe PTSD reaction. It is important, therefore, for leaders and the wider population to encourage a sense of belonging and right to express Jewish identity as a respected group within a diverse and tolerant society. The Royal Commission into Antisemitism and Social Cohesion (https://asc.royalcommission.gov.au/) may play a central role in advancing these objectives.

## 7 Pillar 5: Existential Meaning

Pillar 5, an overarching domain that draws on all four preceding pillars, relates to existential meaning, which is how individuals and communities integrate the terror attack into their existing systems of beliefs and processes of meaning‐making. This is critical to re‐establishing a sense of balance and coherence in the lives of the targeted population. Most survivors will draw on their own strengths and the support provided by significant others to achieve this outcome. This challenge may require a fundamental revision of personal beliefs and priorities in life, a process that can have beneficial outcomes in achieving personal growth and transformations that build resilience against future adversity [3, 11]. For a subgroup, however, a retreat into a profound state of alienation and loss of coherence (which previously was referred to by sociologists as ‘anomie’) can lead to clinical depression and other mental health symptoms, requiring professional help to facilitate recovery [6, 11].

## 8 Conclusion

There will continue to be a multiplicity of mental health and psychosocial interventions offered to survivors and the wider community following the Bondi Beach terror attack, which is appropriate. It is nevertheless important for all contributors to work towards achieving coherence in implementing the overall programme, which will be facilitated by adherence to a common set of principles that provide an overarching conceptual framework to guide the process. Here, we offer an outline of the ADAPT model, which is designed with this aim in that it systematically identifies the essential psychological and social elements of a recovery programme to promote normative adaptive responses in the aftermath of a terrorist event of this type.

Unified leadership, from religious, social and political leaders to front‐line mental health and psychosocial providers, is essential to affirm the shared values underpinning Australian society and is critical to stabilisation following crises of such complexity and magnitude.

## Author Contributions

**Susan J. Rees:** conceptualisation, writing – original draft, writing – review and editing. **Derrick M. Silove:** writing – review and editing.

## Funding

The authors have nothing to report.

## Disclosure

Commissioned; externally peer reviewed.

## Conflicts of Interest

The authors declare no conflicts of interest.

## Data Availability

The authors have nothing to report.
